# Who Has an Unsuccessful Observation Care Stay?

**DOI:** 10.3390/healthcare6040138

**Published:** 2018-11-27

**Authors:** Gelareh Z. Gabayan, Brian Doyle, Li-Jung Liang, Kwame Donkor, David Yu-Chuang Huang, Catherine A. Sarkisian

**Affiliations:** 1 Department of Emergency Medicine, University of California, Los Angeles, CA 90024, USA; kapdonkor@gmail.com; 2 Department of Internal Medicine, Ohio State University, Columbus, OH 43210, USA; doylebr@gmail.com; 3 Divisions of General Internal Medicine and Health Services Research, David Geffen School of Medicine at UCLA, Los Angeles, CA 90095, USA; liangl@ucla.edu (L.-J.L.); YCHuang@mednet.ucla.edu (D.Y.-C.H.); 4 Department of Medicine, Greater Los Angeles Veterans Affairs Healthcare System, Geriatric Research Education and Clinical Center (GRECC), Los Angeles, CA 90073, USA; CSarkisian@mednet.ucla.edu; 5 Department of Medicine, University of California, Los Angeles, CA 90024, USA

**Keywords:** observation care, outcomes, unsuccessful observation care, observation failure

## Abstract

Background: With the recent increase use of observation care, it is important to understand the characteristics of patients that utilize this care and either have a prolonged observation care stay or require admission. Methods: We a conducted a retrospective cohort study utilizing 5% sample data from Medicare patients age ≥65 years that was nationally representative in the year 2013. We performed a generalized estimating equation (GEE) logistic regression analysis to evaluate the relationship between an unsuccessful observation stay (defined as either requiring an inpatient admission from observation or having a prolonged observation stay) compared to having successful observation care. Observation cut offs of “successful” vs. “unsuccessful” were based on the CMS 2 midnight rule. Results: Of 154,756 observation stays in 2013, 19 percent (n = 29,604) were admitted to the inpatient service and 34,275 (22.2%) had a prolonged observation stay. The two diagnoses most likely to have an unsuccessful observation stay were intestinal infections (OR 1.56, 95% CI 1.32–1.83) and pneumonia (OR 1.26, 95% CI 1.13–1.41). Conclusion: We found patients placed in observation care with intestinal infections and pneumonia to have the highest odds of either being admitted from observation or having a prolonged observation stay.

## 1. Introduction

In recent years, there has been greater use of observation services for patients by all types of providers [1,2,3] This care provides a short-term (24–72 h) treatment and assessment, is billed as an outpatient visit, and can take place in the emergency department, inpatient units, special observation units, or any other monitored settings [4] It is utilized by providers to “observe” patients in a monitored setting, usually a hospital. Patients placed in observation care are not well enough to be discharged home and not sick enough to require a prolonged admission. Due to the nature of observation care, patients placed in this care are not expected to require prolonged monitored care.

While the idea of observing a patient dates back to Hippocrates, the increased use of observation care in the US is relatively new [5]. As providers better understand the roles and uses of observation care stays, they require an improved understanding of the outcomes of patients placed in observation care. For inpatient providers and hospital administrators, patients who have unsuccessful observation stays either require an inpatient admission or to have a prolonged observation stay. It is important for both providers and administrators to understand the characteristics of these patients as unsuccessful observation stays are costly to the system, not clinically expected, and may result in unnecessary care. Currently, there are no known studies that assess the characteristics of patients who have an unsuccessful observation stay.

We evaluated 154,756 patients with Medicare Insurance age ≥65 years placed in any US hospital observation care in 2013. The objective of the study was to evaluate the characteristics of patients who utilize observation care and subsequently have an unsuccessful stay, either by being admitted to the inpatient service or by having a prolonged observation stay, defined as ≥2 midnights.

## 2. Methods

### 2.1. Study Design

We performed a retrospective cohort study of a 5% sample of Medicare patients that was nationally representative. All patients were placed in observation care in 2013. The IRB at the University of California, Los Angeles approved the study.

### 2.2. Setting and Selection of Participants

Participants in the study were age ≥65 years at the time of their first day of observation care use. If participants had multiple observation care stays, then only the first stay of the year was included in the analytic sample. Patients who had an observation stay more than 30 days or who were deceased during the observation stay were excluded.

### 2.3. Data Sources

Visit records used for the study analysis were obtained from the Center for Medicare and Medicaid (CMS) Outpatient File, the CMS Inpatient MEDPAR (Medicare Provider Analysis and Review) file, the Master Beneficiary File, and the Chronic Conditions file for 2013.

### 2.4. Measures

Patient comorbidities were derived through the CMS Chronic Conditions file which was linked to the visit records using Claim ID. The CMS Chronic Conditions file contains information regarding the sum total of chronic conditions prior to the observation stay (0–27). Medical diagnoses were obtained based on an algorithm developed by the PI of the study [6,7,8]. In brief, a cross-walk mapping process was linked to the primary ICD-9 code for each observation stay through use of the Multi-level Clinical Classification system (CCS) codes provided by the Healthcare Cost and Utilization Project (HCUP) [9]. The PI developed a total of 39 categories, which have been outlined in the Appendix A. Having used the emergency department (ED) immediately prior to the observation stay, inpatient admission, and use of a skilled nursing facility (SNF) were determined based on Revenue Center Codes as well as charges made to Medicare.

Observation care cut-offs (successful vs. unsuccessful) were based on the CMS 2 midnight rule billing criteria [10] as well as discussion with a set of hospital administrators and inpatient physicians at UCLA and other hospitals. The terms “successful” vs. “unsuccessful” were also obtained through discussion with the administrators and providers. “Unsuccessful” was defined as having at least 2 midnight observation stays or being transferred to the inpatient service. Observation stays of 0 days required at least an 8 h placement in observation care to be billed as “observation”. Each day of observation care usage (i.e., 1 or 30) required the same number of midnights as days.

### 2.5. Data Analysis

Patient characteristics (demographic and clinical) as well as the diagnoses were summarized for the two clinical outcomes following an observation stay (successful observation care stay and unsuccessful observation care stay). In addition, both descriptive statistics and frequency distributions for continuous and categorical variables were generated.

Candidate factors included demographic characteristics, patient comorbidities proxied by the number of CMS chronic conditions, and observation care diagnoses. Clinical Outcomes were modeled using a Generalized Estimating Equation (GEE) logistic regression [11]. The model included all candidate factors as fixed effects and provider-level random effects that accounted for multiple observations within providers.

The model evaluated the factors associated with unsuccessful observation care (inpatient admission from observation care or observation care 2–30 days) vs. successful observation care (0 or 1 days/midnights of observation care which equates to a maximum of 47 h and 59 min). Adjusted odds ratios (AOR) and 95% confidence interval estimates were generated from this analysis. The reference groups for all analyses were the following: Age 65–69, female gender, weekday initial observation placement, observation placement from a non-ED, never used a SNF, no chronic conditions, and observation care diagnosis of “Urinary Tract Infection”. In addition, the study group conducted additional sensitivity analyses regarding patients who attended the ED and weekend vs. weekday visits.

## 3. Results

### 3.1. Sample Characteristics

Table 1 describes the characteristics of the sample. There were close to twice the number of female patients as compared to male (96,742 vs. 57,994). Fifteen percent of the cohort were placed in observation on the weekend and over half of the number of patients placed in observation came from the emergency department. Of all patients placed in observation care, a total of 64,215 (41.5%) came from the ED on a weekday and 21,500 (13.9%) came from the ED on a weekend day. Table 2 describes the diagnoses of the patients with observation stays and their outcomes. Of all diagnoses, diseases of the musculoskeletal system resulted in the highest number of patients placed in observation care (N = 17,401). This diagnosis also had the highest percent of placement (76.3%) in successful observation care. The diagnosis with the greatest number of admissions from observation was pneumonia (1077/1857, 58%). The diagnosis with the greatest percent of prolonged observation was abdominal pain (36.1%).

### 3.2. Main Results

**Figure A1:**
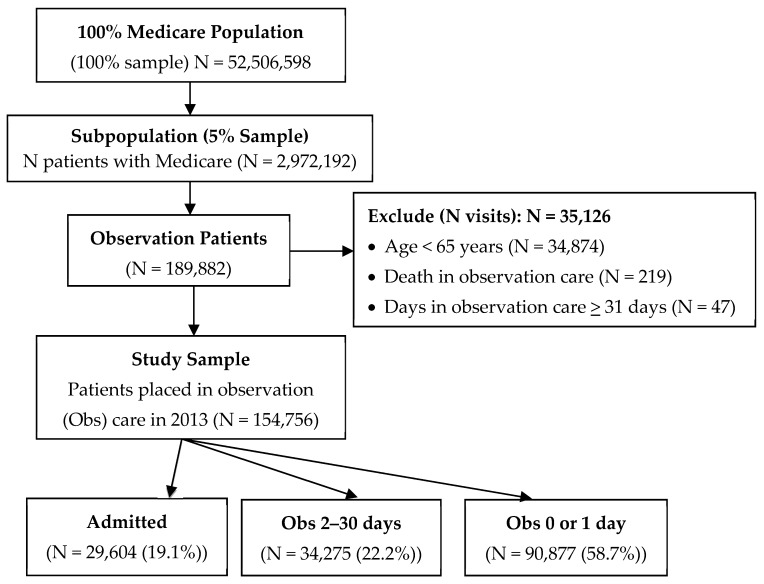
Study Cohort. The original sample of patients with Medicare insurance in 2013 is 52,506,598 individuals. The “Subpopulation” is the 5% sample distributed by the Centers for Medicare and Medicaid (N = 2,972,192). Of the subpopulation, patients in observation care were selected. Of the patients in observation care, the study sample was selected following the application of the exclusion criteria. Of the study sample patients, 19.1% were admitted to the hospital, 22.2% had an observation stay of 2–22 days, and 58.7% had an observation stay of 0–1 days.

Figure A1 (Appendix A) describes the creation of the study cohort. There were 154,756 with an initial observation stay in 2013. Of the cohort placed in observation, 29,604 (19.1%) were admitted to the inpatient service and 34,275 (22.2%) had a prolonged observation stay. Table 3 describes the GEE results of the model assessing the factors associated with an unsuccessful observation stay (admission or >2 days) vs. successful observation care (0–1 days). The top two diagnoses most likely to have an unsuccessful observation stay were intestinal infections (AOR 1.56, 95% CI 1.32–1.83) and pneumonia (AOR 1.26, 95% CI 1.13–1.41). Patients placed in observation care on a weekend (AOR 1.28, 95% CI 1.24–1.32), came from the emergency department (AOR 2.84, 95% CI 2.74–2.95) or utilized a skilled nursing facility (AOR 2.85, 95% CI 2.68–3.02) also had high odds of an unsuccessful observation stay.

## 4. Discussion

In recent years, there has been a greater use of observation care [1,2,12,13] This type of “temporary” care allows providers to place patients in a monitored setting, usually a hospital, where they can be watched for 0–48 h while being considered an outpatient encounter [5] For providers, administrators, and health policy experts, it is important to understand the type of patients that have an unsuccessful observation stay, defined as either having a prolonged observation stay or getting admitted from observation care, as having an unsuccessful observation care stay is not only unexpected to the health care system but it may result in greater cost and unnecessary care for the system. We found that patients with intestinal infections and pneumonia have the highest likelihood of having an unsuccessful observation care stay. In addition, we also found that patients coming from the ED, seen on a weekend as compared to weekday, and having been placed in a skilled nursing facility to have a higher rate of an unsuccessful observation stay.

The diagnosis with the highest odds of having an unsuccessful observation care stay was an intestinal infection, ranging from a rare diagnosis such as Cholera or Shigella to an ill-defined diagnosis. An intestinal infection is commonly a condition that is transitory in nature and while physically uncomfortable, less likely to require aggressive treatment. The findings of this study suggest that if a patient requires placement in the hospital, there may be additional factors not identifiable in administrative data that could lead to prolonged care such as dehydration and/or requirement of an extended course of treatment.

Pneumonia had the second highest odds of an unsuccessful observation care stay. Over 50% of pneumonias are classified as community acquired pneumonia [14]. While the epidemiology and bacteriology of all the types of pneumonia are different, on initial presentation a provider is unable to distinguish between the different kinds of pneumonia until further testing is done [15]. As pneumonia is an infection that can have an unpredictable course, it is understandable that patients with pneumonia had a high rate of an unsuccessful observation care stay. It is also possible that patients with pneumonia were misdiagnosed.

We found that originating from the emergency department had a high odds of an unsuccessful observation care stay. Patients placed in observation care can range between having come from an acute encounter or a scheduled procedure and providers in the ED often lack historical information on patients [5]. The unpredictability of the type of patents presenting to the ED as well as the lack of history may lead to ED providers not understanding the complexity of care patients may need. It is important for health care administrators to be aware of these finding so that if patients do originate from the ED, they receive a more defined method of management.

Patients placed in observation care on a weekend had a higher likelihood of an unsuccessful observation care stay. This could be a result of multiple factors. Care delivered to patients on weekends does not often include the complete staff and services needed. In addition, patients may have prolonged seeing a provider until the weekend and the condition could have worsened. Although the study controlled for number of comorbidities and conditions, it was unable to account for severity of illness.

Patients in a skilled nursing facility (SNF) usually have a greater number of medical problems and require more ancillary care [16] As these patients are more “complex” it would be expected that they would have a greater likelihood of having a prolonged observation stay or requiring admission following their observation care. In the same light, it would lead to an excess in resource utilization if all patients from a SNF were admitted. Providers seeing these patients should continue to evaluate and develop a disposition plan based on need but should keep in mind that these patients have a higher likelihood of not being successful in their observation care stay.

### Limitations

The study has some limitations. First, the analysis is based on data derived from claim ID, billing data, and ICD-9 codes, which are limited in that they are retrospective and can reflect incomplete coding. Second, a majority of patients who use Medicare insurance do not visit Federal hospitals, so these findings are not generalizable to Federal facilities [3]. Third, the analysis did not include information from prior year observation stays as that would require use of data from a prior year that the team did not have. Also, the files lack clinical variables such as vital signs and physical exam. The files also lack information regarding hospital characteristics such as teaching vs. non, rural vs. non, average income of hospitals, etc. Finally, the data is several years old as a result of the time it took to acquire (2 years), link and clean the files (2 years). Despite these limitations, this study provides important information regarding older Medicare beneficiaries that experience observation stay.

## 5. Conclusions

With the rise of observation care utilization, we assessed the factors associated with having an unsuccessful observation care stay. Patients with either an intestinal infection or pneumonia had the highest odds of an unsuccessful observation care stay. In addition, patients coming from the emergency department, placed in observation care on a weekend, or requiring a skilled nursing facility had the highest likelihood of lack of observation success. This study provides relevant and essential information for both providers and hospital administrators.

## Figures and Tables

**Table 1:** Observation sample characteristics.

Characteristic	Total (N)	Admitted N (%)	OBS 2–30 Days N (%)	OBS 0 or 1 Day N (%)
**Age ^1^**				
65–69	31,219	4636 (14.9)	5983 (19.2)	20,600 (65.9)
70–74	30,182	4954 (16.4)	5986 (19.8)	19,242 (63.8)
75–79	29,487	5583 (18.9)	6368 (21.6)	17,536 (59.5)
80+	63,866	14,431 (22.6)	15,938 (25.0)	33,499 (52.4)
**Gender**				
Female	96,762	18,567 (19.2)	22,577 (23.3)	55,618 (57.5)
Male	57,994	11,037 (19.0)	11,698 (20.2)	35,259 (60.8)
**Race/Ethnicity ^4^**				
White	134,753	25,158 (18.7)	29,317 (21.8)	80,278 (59.6)
lack	13,215	3045 (23.0)	3421 (25.9)	6749 (51.1)
Asian	1885	414 (22.0)	420 (22.3)	1051 (55.8)
Hispanic	2156	538 (25.0)	547 (25.4)	1071 (49.7)
North American N	645	92 (14.3)	168 (26.0)	385 (59.7)
**Day of week of service**				
Weekday	131,486	22,631 (17.2)	27,549 (21.0)	81,306 (61.8)
Weekend	23,270	6973 (30.0)	6726 (28.9)	9571 (41.1)
**Observation care from an ED**				
NO	69,041	4001 (5.8)	11,286 (16.3)	53,754 (77.9)
YES	85,715	25,603 (29.9)	22,989 (26.8)	37,123 (43.3)
**SNF ^2^ utilization**				
NO	74,420	1 (0)	17,045 (22.9)	57,374 (77.1)
YES	80,336	29,603 (36.8)	17,230 (21.5)	33,503 (41.7)
**Comorbidity ^3^**				
Acute Myocardial Infarction	12,860	2932 (22.8)	3108 (24.2)	6820 (53.0)
Alzheimer’s Disease	12,844	3113 (24.2)	3721 (29.0)	6010 (46.8)
Alzheimer’s Disease and Related Disorders	32,060	7578 (23.6)	9106 (28.4)	15,376 (48.0)
Atrial Fibrillation	36,946	7815 (21.2)	9088 (24.6)	20,043 (54.2)
Cataract	109,907	19,547 (17.8)	25,474 (23.2)	64,886 (59.0)
Chronic Kidney Disease	55,218	11,993 (21.7)	13,873 (25.1)	29,352 (53.2)
Chronic Obstructive Pulmonary Disease	56,578	12,029 (21.3)	14,175 (25.1)	30,374 (53.7)
Heart Failure	62,989	14,094 (22.4)	16,040 (25.5)	32,855 (52.2)
Diabetes	66,402	13,334 (20.1)	16,143 (24.3)	36,925 (55.6)
Glaucoma	37,932	6681 (17.6)	8980 (23.7)	22,271 (58.7)
Hip/Pelvic Fracture	9112	2119 (23.3)	2456 (27.0)	4537 (49.8)
Ischemic Heart Disease	97,143	19,525 (20.1)	23,272 (24.0)	54,346 (55.9)
Depression	59,719	11,590 (19.4)	14,993 (25.1)	33,136 (55.5)
Osteoporosis	43,268	8067 (18.6)	10,805 (25.0)	24,396 (56.4)
Rheumatoid Arthritis/Osteoarthritis	101,301	18,242 (18.0)	24,036 (23.7)	59,023 (58.3)
Stroke/Transient Ischemic Attack	35,114	7997 (22.8)	9170 (26.1)	17,947 (51.1)
Breast Cancer	12,449	1843 (14.8)	2939 (23.6)	7667 (61.6)
Colorectal Cancer	6647	1212 (18.2)	1620 (24.4)	3815 (57.4)
Prostate Cancer	10,135	1663 (16.4)	2074 (20.5)	6398 (63.1)
Lung Cancer	4644	789 (17.0)	1119 (24.1)	2736 (58.9)
Endometrial Cancer	2167	345 (15.9)	539 (24.9)	1283 (59.2)
Anemia	100,552	19,592 (19.5)	24,596 (24.5)	56,364 (56.1)
Asthma	27,545	5612 (20.4)	6807 (24.7)	15,126 (54.9)
Hyperlipidemia	125,221	22,660 (18.1)	28,804 (23.0)	73,757 (58.9)
Benign Prostatic Hyperplasia	30,077	5293 (17.6)	6521 (21.7)	18,263 (60.7)
Hypertension	134,494	25,096 (18.7)	31,324 (23.3)	78,074 (58.1)
Acquired Hypothyroidism	47,856	9040 (18.9)	11,673 (24.4)	27,143 (56.7)

^1^ Age at observation admission. ^2^ Skilled Nursing Facility utilization in 2013. ^3^ Comorbidity based on the CMS Chronic Conditions. ^4^ Of race/ethnicity was, 1% was reported as “Other” and 0.4% was unknown.

**Table 2:** Observation sample diagnoses (N = 154,756).

Characteristic	Total (N = 154,756)	Obs 0–1 Day (N = 90,877)	Admitted (N = 29,604)	Obs 2–30 Days (N = 34,275)
	N (%)	N (%)	N (%)	N (%)
Diseases of the musculoskeletal system skin and connective tissue	17,401 (11.2)	13,278 (76.3)	1095 (6.3)	3028 (17.4)
Chest pain	15,202 (9.8)	11,283 (74.2)	707 (4.7)	3212 (21.1)
Neoplasms	12,298 (7.9)	9142 (74.3)	840 (6.8)	2316 (18.8)
GI System Diseases	9932 (6.4)	4295 (43.2)	3120 (31.4)	2517 (25.3)
Dizziness vertigo and syncope	7439 (4.8)	4244 (57.1)	689 (9.3)	2506 (33.7)
Other Residual codes	6823 (4.4)	5117 (75)	302 (4.4)	1404 (20.6)
Dysrhythmias and condition disorders	6169 (4)	3430 (55.6)	1639 (26.6)	1100 (17.8)
Nervous System Disorders	5725 (3.7)	3935 (68.7)	728 (12.7)	1062 (18.6)
Ischemic Heart Disease	5346 (3.5)	2421 (45.3)	2055 (38.4)	870 (16.3)
Endocrine nutritional immunity and metabolic disorders	5066 (3.3)	2782 (54.9)	984 (19.4)	1300 (25.7)
Other Renal and GU Diseases	4941 (3.2)	3572 (72.3)	436 (8.8)	933 (18.9)
Circulatory Disorders: Disease of arteries arterioles vei	4547 (2.9)	2420 (53.2)	910 (20)	1217 (26.8)
Minor Injuries	4150 (2.7)	1568 (37.8)	1206 (29.1)	1376 (33.2)
Cerebrovascular Disease	3789 (2.4)	1422 (37.5)	1575 (41.6)	792 (20.9)
Other Injuries	3666 (2.4)	2201 (60)	301 (8.2)	1164 (31.8)
Other Respiratory Disease	3240 (2.1)	2182 (67.3)	439 (13.5)	619 (19.1)
Urinary Tract Infection	3218 (2.1)	1014 (31.5)	1320 (41)	884 (27.5)
Diseases of the blood	3122 (2)	2007 (64.3)	462 (14.8)	653 (20.9)
Chronic obstructive pulmonary disease COPD	3045 (2)	1130 (37.1)	1180 (38.8)	735 (24.1)
Congestive Heart Failure	2994 (1.9)	871 (29.1)	1476 (49.3)	647 (21.6)
Complications and Adverse events	2958 (1.9)	1363 (46.1)	922 (31.2)	673 (22.8)
Other Symptoms	2699 (1.7)	1512 (56)	252 (9.3)	935 (34.6)
Hypertension HTN	2459 (1.6)	1581 (64.3)	421 (17.1)	457 (18.6)
Diabetes with and without complications	2455 (1.6)	1509 (61.5)	360 (14.7)	586 (23.9)
Other Infectious and Parasitic Diseases	2343 (1.5)	954 (40.7)	1166 (49.8)	223 (9.5)
Pneumonia	1857 (1.2)	444 (23.9)	1077 (58)	336 (18.1)
Abdominal pain	1644 (1.1)	914 (55.6)	137 (8.3)	593 (36.1)
Renal Disease	1642 (1.1)	471 (28.7)	899 (54.8)	272 (16.6)
Mental Illness	1592 (1)	730 (45.9)	384 (24.1)	478 (30)

Total sample included all patients in the study cohort: Row percents are presented. Patients with a <1% diagnosis not included.

**Table 3:** GEE logistic regression for unsuccessful observation care stay.

Patient Characteristics	Odds Ratio (95% CI)	*p*
**Age (REF = 65–69)**		
70–74	1.05 (1.01–1.09)	0.0066
75–79	1.14 (1.1–1.18)	<0.0001
80+	1.23 (1.19–1.27)	<0.0001
**Gender**		
Male vs. Female	0.92 (0.9–0.94)	<0.0001
**Race/Ethnicity (REF = White)**		
Black	1.22 (1.17–1.27)	<0.0001
Others	1.06 (0.97–1.15)	0.2049
Asian/PI	1.17 (1.05–1.31)	0.0051
Hispanic	1.11 (1.01–1.22)	0.036
**Day of week of service**		
Weekend vs. Weekday	1.28 (1.24–1.32)	<0.0001
**Observation care from an ED visit**		
Yes vs. No	2.84 (2.74–2.95)	<0.0001
**Ever used SNF services in 2013**		
Yes vs. No	2.85 (2.68–3.02)	<0.0001
Number of chronic conditions ^1^	0.98 (0.98–0.99)	<0.0001
**Observation diagnosis (REF = Urinary Tract Infection)**		
Intestinal Infection	1.56 (1.32–1.83)	<0.0001
Pneumonia	1.26 (1.13–1.41)	<0.0001
Other Infectious and Parasitic Diseases ^2^	1.13 (1.01–1.27)	0.0278
Renal Disease	1.08 (0.96–1.23)	0.2008
Skin and Subcutaneous Infections	1.04 (0.93–1.18)	0.4759
CHF	0.97 (0.88–1.07)	0.5597
Asthma	0.96 (0.82–1.13)	0.6567
Minor Injuries	0.86 (0.79–0.94)	0.0009
GI system Diseases	0.83 (0.76–0.89)	<0.0001
COPD	0.82 (0.75–0.91)	<0.0001
Non-atherosclerotic Heart Disease	0.79 (0.68–0.91)	0.0012
Non-infectious Lung Disease	0.76 (0.65–0.88)	0.0004
Complications and Adverse events	0.75 (0.68–0.83)	<0.0001
Ischemic Heart Disease	0.73 (0.67–0.81)	<0.0001
Circulatory Disorders	0.73 (0.66–0.81)	<0.0001
Cerebrovascular Diseases	0.72 (0.66–0.79)	<0.0001
Mental Illness	0.65 (0.57–0.74)	<0.0001
Upper Respiratory Infection	0.64 (0.56–0.72)	<0.0001
Diabetes Mellitus	0.62 (0.55–0.7)	<0.0001
Endocrine, nutritional, immunity and metabolic disorders	0.6 (0.55–0.65)	<0.0001
Neoplasms	0.59 (0.54–0.66)	<0.0001
Other Renal and GI Diseases	0.58 (0.52–0.64)	<0.0001
Dysrhythmias	0.53 (0.49–0.59)	<0.0001
Congenital Diseases	0.53 (0.34–0.83)	0.0058
Major Injuries	0.52 (0.43–0.63)	<0.0001
Nervous system Disorders	0.51 (0.46–0.56)	<0.0001
Other Injuries	0.49 (0.44–0.55)	<0.0001
Diseases of the musculoskeletal system, skin and connective tissue	0.49 (0.45–0.53)	<0.0001
Hypertension	0.48 (0.43–0.54)	<0.0001
Symptoms: Abdominal Pain	0.47 (0.42–0.54)	<0.0001
Symptoms: Others	0.47 (0.42–0.51)	<0.0001
Diseases of the blood	0.45 (0.4–0.51)	<0.0001
Other Residual Codes	0.42 (0.38–0.47)	<0.0001
Symptoms: Dizziness, Vertigo and Syncope	0.38 (0.35–0.42)	<0.0001
Other Respiratory Diseases	0.38 (0.34–0.42)	<0.0001
Symptoms: Headache	0.32 (0.26–0.41)	<0.0001
Symptoms: Chest Pain	0.17 (0.16–0.19)	<0.0001

Unsuccessful Observation Care Stay defined as an observation stay that resulted in Admission or a prolonged Observation stay defined as a stay 2–30 days. ^1^ Number of CMS Chronic Conditions based on 0–27 conditions. ^2^ Including Meningitis, Infective Arthritis, Bacterial, Mycoses, Viral.

## Appendix A

**Table A1:** CMS chronic Conditions.

Name of Chronic Condition	Variable Name in the Dataset
Acute Myocardial Infarction	AMIc
Alzheimer’s Disease	ALZHc
Alzheimer’s Disease and Related Disorders	ALZH_DEMENc
Atrial Fibrillation	ATRIAL_FIBc
Cataract	CATARACTc
Chronic Kidney Disease	CHRONICKIDNEYc
Chronic Obstructive Pulmonary Disease	COPDc
Heart Failure	CHFc
Diabetes	DIABETESc
Glaucoma	GLAUCOMAc
Hip/Pelvic Fracture	HIP_FRACTUREc
Ischemic Heart Disease	ISCHEMICHEARTc
Depression	DEPRESSIONc
Osteoporosis	OSTEOPOROSISc
Rheumatoid Arthritis/Osteoarthritis	RA_OAc
Stroke/Transient Ischemic Attack	STROKE_TIAc
Breast Cancer	CANCER_BREASTc
Colorectal Cancer	CANCER_COLORECTALc
Prostate Cancer	CANCER_PROSTATEc
Lung Cancer	CANCER_LUNGc
Endometrial Cancer	CANCER_ENDOMETRIALc
Anemia	ANEMIAc
Asthma	ASTHMAc
Hyperlipidemia	HYPERLc
Benign Prostatic Hyperplasia	HYPERPc
Hypertension	HYPERTc
Acquired Hypothyroidism	HYPOTHc

**Table A2:** Diagnosis codes.

Diagnosis	Codes
Injuries: Sprains, fractures and joint disorders	16.1 16.2 16.7
Injuries: Major trauma related: Spinal cord, Intracranial, Crushing/internal organ injury	16.3 16.4 16.5
Injuries: Other including burns, wounds, poisonings, superficial injuries	16.6 16.8 16.9 16.11 16.12
Symptoms: Abdominal pain	17.1.7
Symptoms: Chest pain	7.2.5
Symptoms: Dizziness, vertigo and syncope	6.8.2 17.1.1
Symptoms: Headache	6.5
Symptoms: Other symptoms, signs and ill-defined conditions	17.1.2 17.1.3 17.1.4 17.1.5 17.1.6 17.1.8 17.1.9
Infection: Upper respiratory infections excluding pneumonia	8.1.2 8.1.3 8.1.4 8.1.5
Infection: Intestinal Infections	9.1
Infection: Urinary Tract infection and symptoms	10.1.4
Infection: Other Infectious and Parasitic Diseases: Meningitis, Infective arthritis, Bacterial, Mycoses, Viral	1 6.1 13.1
Infection: Skin and SubQ Infection	12.1
Endocrine; nutritional; and metabolic diseases and immunity disorders	3.1 3.4 3.5 3.6 3.7 3.8 3.9 3.10 3.11
Diabetes with and without complications	3.2 3.3
HTN	7.1
Other Heart Disease: Valvular disease, Carditis	7.2.1 7.2.2 7.2.6 7.2.7 7.2.10
Dysrythmias and conduction disorders	7.2.8 7.2.9
Ischemic Heart Disease and MI	7.2.3 7.2.4
CHF	7.2.11
Circulatory Disorders: Diseases of arteries; arterioles; veins; lymphatics and capillaries	7.4 7.5
Cerebrovascular Disease	7.3
Diseases of the blood and blood-forming organs	4
Neoplasms	2
Mental Illness	5
Nervous System Disorders	6.2 6.3 6.4 6.6 6.7 6.8.1 6.8.3 6.9
Pneumonia	8.1.1
Other Respiratory Disease	8.6 8.7 8.8 8.9
COPD	8.2
Asthma	8.3
Pleurisy, Pneumothorax, and Pneumonitis	8.4 8.5
GI System Diseases	9.2 9.3 9.4 9.5 9.6 9.7 9.8 9.9 9.10 9.11 9.12
Other Renal and GU Diseases	10.1.5 10.1.6 10.1.7 10.2 10.3 10.1.8
Renal Disease	10.1.1 10.1.2 10.1.3
Pregnancy and childbirth related disorders	11
Congenital and Perinatal Anomalies	14 15
Diseases of the musculoskeletal system, skin and connective tissue	12.2 12.3 12.4 13.2 13.3 13.4 13.5 13.6 13.7 13.8 13.9
Complications and Adverse events	16.10
Other: Residual codes and other factors influencing healthcare	17.2 18

Based on the Clinical Classification Software (CCS) Multilevel ICD-9 codes devised by the Healthcare Cost and Utilization Project (HCUP).

## References

[1] Cafardi S.G., Pines J.M., Deb P., Powers C.A., Shrank W.H. (2016). Increased observation services in Medicare beneficiaries with chest pain. Am. J. Emerg. Med..

[2] Feng Z., Wright B., Mor V. (2012). Sharp rise in Medicare enrollees being held in hospitals for observation raises concerns about causes and consequences. Health Aff..

[3] Wright B., O’Shea A.M., Ayyagari P., Ugwi P.G., Kaboli P., Vaughan Sarrazin M. (2015). Observation rates at veterans’ hospitals more than doubled during 2005–13, similar to medicare trends. Health Aff..

[4] Centers for Medicare & Medicaid Services (2011). Part B hospital (including inpatient hospital Part B and OPPS). Medicare Claims Processing Manual.

[5] Aston G. (2012). Observation units: A tightrope act. Hosp. Health Netw..

[6] Gabayan G.Z., Derose S.F., Asch S.M., Yiu S., Lancaster E.M., Poon K.T., Hoffman J.R., Sun B.C. (2011). Patterns and predictors of short-term death after emergency department discharge. Ann. Emerg. Med..

[7] Gabayan G.Z., Asch S.M., Hsia R.Y., Zingmond D., Liang L., Han W., McCreath H., Weiss R.E., Sun B.C. (2013). Factors associated with short-term bounce-back admissions following emergency department discharge. Ann. Emerg. Med..

[8] Gabayan G.Z., Sarkisian C.A., Liang L.J., Sun B.C. (2015). Predictors of admission after emergency department discharge in older adults. J. Am. Geriatr. Soc..

[9] Clinical Classification Software (CCS) for ICD-9-CM. https://www.hcup-us.ahrq.gov/toolssoftware/ccs/ccs.jsp.

[10] Centers for Medicare & Medicaid Services Fact Sheet: Two-Midnight Rule. https://www.cms.gov/Newsroom/MediaReleaseDatabase/Fact-sheets/2015-Fact-sheets-items/2015-07-01-2.html.

[11] Diggie P.J., Heagerty P., Liang K.Y., Zeger S.L. (2002). Analysis of Longitudinal Data.

[12] Baugh C.W., Venkatesh A.K., Hilton J.A., Samuel P.A., Schuur J.D., Bohan J.S. (2012). Making greater use of dedicated hospital observation units for many short-stay patients could save $3.1 billion a year. Health Aff..

[13] Blecker S., Gavin N.P., Park H., Ladapo J.A., Katz S.D. (2016). Observation units as substitutes for hospitalization or home discharge. Ann. Emerg. Med..

[14] Abrahamian F.M., Deblieux P.M., Emerman C.L., Kollef M.H., Kupersmith E., Leeper K.V., Paterson D.L., Shorr A.F. (2008). Health care-associated pneumonia: identification and initial management in the ED. Am. J. Emerg. Med..

[15] Kollef M.H., Shorr A., Tabak Y.P., Gupta V., Liu L.Z., Johannes R.S. (2005). Epidemiology and outcomes of health-care-associated pneumonia: Results from a large US database of culture-positive pneumonia. Chest.

[16] Shaughnessy P.W., Kramer A.M. (1990). The increased needs of patients in nursing homes and patients receiving home health care. N. Engl. J. Med..

